# Structural polymorphism in the L1 loop regions of human H2A.Z.1 and H2A.Z.2

**DOI:** 10.1107/S090744491302252X

**Published:** 2013-11-19

**Authors:** Naoki Horikoshi, Koichi Sato, Keisuke Shimada, Yasuhiro Arimura, Akihisa Osakabe, Hiroaki Tachiwana, Yoko Hayashi-Takanaka, Wakana Iwasaki, Wataru Kagawa, Masahiko Harata, Hiroshi Kimura, Hitoshi Kurumizaka

**Affiliations:** a Laboratory of Structural Biology, Graduate School of Advanced Science and Engineering, Waseda University, 2-2 Wakamatsu-cho, Shinjuku-ku, Tokyo 162-8480, Japan; b Graduate School of Frontier Biosciences, Osaka University, 1-3 Yamada-oka, Suita, Osaka 565-0871, Japan; c RIKEN, 2-1 Hirosawa, Wako-shi, Saitama 351-0198, Japan; d Program in Chemistry and Life Science, Department of Interdisciplinary Science and Engineering, School of Science and Engineering, Meisei University, 2-1-1 Hodokubo, Hino-shi, Tokyo 191-8506, Japan; e Laboratory of Molecular Biology, Graduate School of Agricultural Science, Tohoku University, Tsutsumidori-Amamiya-machi 1-1, Aoba-ku, Sendai 981-8555, Japan

**Keywords:** H2A.Z.1, H2A.Z.2, nucleosomes, chromatin, FRAP

## Abstract

The histone H2A.Z variant is widely conserved among eukaryotes. Two isoforms, H2A.Z.1 and H2A.Z.2, have been identified in vertebrates and may have distinct functions in cell growth and gene expression. However, no structural differences between H2A.Z.1 and H2A.Z.2 have been reported. In the present study, the crystal structures of nucleosomes containing human H2A.Z.1 and H2A.Z.2 were determined. The structures of the L1 loop regions were found to clearly differ between H2A.Z.1 and H2A.Z.2, although their amino-acid sequences in this region are identical. This structural polymorphism may have been induced by a substitution that evolutionally occurred at the position of amino acid 38 and by the flexible nature of the L1 loops of H2A.Z.1 and H2A.Z.2. It was also found that in living cells nucleosomal H2A.Z.1 exchanges more rapidly than H2A.Z.2. A mutational analysis revealed that the amino-acid difference at position 38 is at least partially responsible for the distinctive dynamics of H2A.Z.1 and H2A.Z.2. These findings provide important new information for understanding the differences in the regulation and functions of H2A.Z.1 and H2A.Z.2 in cells.

## Abstract 2

The crystal structures of human nucleosomes containing H2A.Z.1 and H2A.Z.2 have been determined. Structural polymorphisms were found in the L1 loop regions of H2A.Z.1 and H2A.Z.2 in the nucleosomes that are likely to be caused by their flexible nature.

## 1. Introduction

In eukaryotes, genomic DNA is organized and compacted as chromatin, in which the DNA is packaged into nucleosomes by histones. In the nucleosome, two of each of the histones H2A, H2B, H3 and H4 form the histone octamer and about 145–147 base pairs of DNA are wrapped around it (Luger *et al.*, 1997 ▶). The nucleosomes are connected by short linker DNA segments and form chromatin fibres with a beads-on-a-­string configuration. The nucleosome–nucleosome inter­actions within or between chromatin fibres contribute to the organization of higher-order chromatin (Luger *et al.*, 2012 ▶).

The canonical histones H2A, H2B, H3 and H4 are mainly incorporated into chromatin during the S-phase of the cell cycle by timely expression (Marzluff *et al.*, 2008 ▶). In addition to the canonical histones, non-allelic histone variants, with production that is not restricted to the S-phase, have also been identified (Talbert *et al.*, 2012 ▶). For H2A, four classes of variants, H2A.B, H2A.X, mH2A and H2A.Z, exist in human somatic cells and have been suggested to play specific roles in the regulation of transcription, replication, recombination and repair of genomic DNA (Bönisch & Hake, 2012 ▶; Millar, 2013 ▶).

Histone H2A.Z, one of the most widely distributed histone variants among eukaryotes, is reportedly involved in many biological processes, such as transcriptional regulation, genome stability, chromosome segregation, cellular proliferation, heterochromatin organization and epigenetic memory regulation, probably by modification of the chromatin structure (Bönisch & Hake, 2012 ▶; Millar, 2013 ▶). Accumulation of H2A.Z at DNA double-strand break sites has also been found (Xu *et al.*, 2012 ▶), suggesting its involvement in DNA repair. H2A.Z is predominantly localized around the promoters of active genes and functions to create nucleosome-free regions near transcription start sites (Guillemette *et al.*, 2005 ▶; Albert *et al.*, 2007 ▶; Barski *et al.*, 2007 ▶; Zilberman *et al.*, 2008 ▶; Cui *et al.*, 2009 ▶; Hardy *et al.*, 2009 ▶; Conerly *et al.*, 2010 ▶; Kelly *et al.*, 2010 ▶; Weber *et al.*, 2010 ▶; Soboleva *et al.*, 2011 ▶; Valdés-Mora *et al.*, 2012 ▶). In fact, the promoter-proximal H2A.Z is dynamically exchanged during the cell cycle (Nekrasov *et al.*, 2012 ▶). H2A.Z also plays essential roles in embryonic stem cells, in which drastic changes of the chromatin states during differentiation are promoted for the recruitment of transcription cofactors (Creyghton *et al.*, 2008 ▶; Li *et al.*, 2012 ▶; Hu *et al.*, 2013 ▶).

Two non-allelic isoforms, H2A.Z.1 and H2A.Z.2, exist in vertebrates (Eirín-López *et al.*, 2009 ▶), and a splicing variant, H2A.Z.2.2, is also expressed from the *H2A.Z.2* gene as a minor or tissue-specific transcript (Bönisch *et al.*, 2012 ▶). In mice, *H2A.Z.1* gene knockout causes lethality, although the *H2A.Z.2* gene is still present, indicating that H2A.Z.1 plays unique roles (Faast *et al.*, 2001 ▶). Chicken DT40 cells lacking either the *H2A.Z.1* or the *H2A.Z.2* gene exhibited distinct alterations in cell growth and gene expression (Matsuda *et al.*, 2010 ▶). In humans, the *H2A.Z.1* and *H2A.Z.2* genes share similar expression profiles in a wide range of tissues, but their distributions in chromatin are different (Dryhurst *et al.*, 2009 ▶). These findings suggest that H2A.Z.1 and H2A.Z.2 function differently in cells. However, the structural basis of the differences between the nucleosomes containing H2A.Z.1 and H2A.Z.2 has not been elucidated owing to a lack of structural information for H2A.Z.2.

In the present study, we determined the crystal structures of human nucleosomes containing H2A.Z.1 and H2A.Z.2, and discovered a structural polymorphism in the L1 loop regions. We also found that the mobilities of H2A.Z.1 and H2A.Z.2 in living cells are different. The structural polymorphism and distinct dynamics of H2A.Z.1 and H2A.Z.2 may be caused by the amino-acid difference at position 38, which is serine in H2A.Z.1 and threonine in H2A.Z.2.

## 2. Materials and methods

### 2.1. Preparation of human nucleosomes containing H2A.Z.1 or H2A.Z.2

Human histones H2B, H3.1 and H4 were prepared by the methods described previously (Tanaka *et al.*, 2004 ▶; Tachiwana *et al.*, 2011 ▶). Human histones H2A.Z.1 and H2A.Z.2 were bacterially expressed and were purified by the same method as used for canonical histone H2A (Tachiwana *et al.*, 2011 ▶). To reconstitute the histone octamers containing either H2A.Z.1 or H2A.Z.2, purified H2B, H3.1, H4 and H2A.Z.1 or H2A.Z.2 were mixed in 20 m*M* Tris–HCl buffer pH 7.5 containing 7 *M* guanidine hydrochloride and 20 m*M* β-mercaptoethanol and rotated at 277 K for 1.5 h. The samples were dialyzed against 10 m*M* Tris–HCl buffer pH 7.5 containing 1 m*M* EDTA, 2 *M* NaCl and 5 m*M* β-mercaptoethanol. The resulting histone octamers were fractionated by HiLoad 16/60 Superdex 200 gel-­filtration column chromatography (GE Healthcare Life Sciences) in 10 m*M* Tris–HCl buffer pH 7.5 containing 1 m*M* EDTA, 2 *M* NaCl and 5 m*M* β-mercaptoethanol. The purified histone octamers containing either H2A.Z.1 or H2A.Z.2 were mixed with the 146-base-pair DNA in a solution containing 2 *M* KCl, and the KCl concentration was gradually decreased to 0.25 *M* during dialysis. The samples were finally dialyzed against 10 m*M* Tris–HCl buffer pH 7.5 containing 1 m*M* EDTA, 1 m*M* dithiothreitol and 250 m*M* KCl at 277 K for 4 h. To remove nonspecific histone–DNA aggregates, the samples were incubated at 328 K for 2 h. The H2A.Z.1 and H2A.Z.2 nucleosomes were further purified by nondenaturing polyacrylamide gel electrophoresis using a Prep Cell apparatus (Bio-Rad). For crystallization, the purified nucleosomes were concentrated and dialyzed against 20 m*M* potassium cacodylate buffer pH 6.0 containing 1 m*M* EDTA.

### 2.2. Crystallization and structure determination

The crystals of human nucleosomes containing H2A.Z.1 and H2A.Z.2 were grown by the hanging-drop method at 293 K. The hanging drop was formed by adding 1 µl of each nucleosome (at a concentration of 2.5–4.0 mg ml^−1^) to 1 µl crystallization solution (20 m*M* potassium cacodylate pH 6.0, 50–70 m*M* KCl, 75–105 m*M* MnCl_2_). For data collection, the crystals were harvested in reservoir solution containing 30% 2-methyl-2,4-pentanediol and 2% trehalose and were flash-cooled in a stream of nitrogen gas at 100 K. Data sets were collected on the BL41XU and BL38B1 beamlines at SPring-8, Harima, Japan. The data sets were processed and scaled using the *HKL*-2000 program suite (Otwinowski & Minor, 1997 ▶). All nucleosome crystals belonged to the orthorhombic space group *P*2_1_2_1_2_1_ and contained one nucleosome per asymmetric unit. Unit-cell parameters are provided in Table 1 ▶.

The structures of the H2A.Z.1 and H2A.Z.2 nucleosomes were solved to 3.07 and 3.20 Å resolution, respectively. The data were processed using the *CCP*4 suite of programs (Winn *et al.*, 2011 ▶). The structures of the nucleosomes were determined by molecular replacement with *Phaser* (McCoy *et al.*, 2007 ▶) using the coordinates of the human nucleosome structure (PDB entry 3afa; Tachiwana *et al.*, 2010 ▶) as the search model. To eliminate the model bias from the electron-density map, we removed the Lys36–Arg42 region of H2A from the model prior to the initial round of refinement. All refinements were performed using *PHENIX* (Adams *et al.*, 2010 ▶). After rigid-body refinement, the model was refined by iterative rounds of *xyz*-coordinate, real-space, individual *B*-­factor and occupancy refinements and manual model building using *Coot* (Emsley & Cowtan, 2004 ▶). For all refinements, secondary-structure restraints and noncrystallographic symmetry (NCS) restraints were applied between chains *A* and *E*, chains *B* and *F*, chains *C* and *G* and chains *D* and *H*. The Ramachandran plots of the final structures showed no outlying residues, as assessed with the *MolProbity* program (Chen *et al.*, 2010 ▶). A summary of the data-collection and refinement statistics is provided in Table 1 ▶. All structure figures were created using *PyMOL* (Schrödinger; http://www.pymol.org). The atomic coordinates of all nucleosomes have been deposited in the RCSB Protein Data Bank, with codes 3wa9 and 3waa for the H2A.Z.1 and H2A.Z.2 nucleosomes, respectively.

### 2.3. R.m.s.d. and *B*-factor calculations

Each pair of crystal structures of the H2A and H2A.Z.1 nucleosomes, the H2A and H2A.Z.2 nucleosomes or the H2A.Z.1 and H2A.Z.2 nucleosomes was superimposed and the r.m.s.d. value for each C^α^ atom was calculated using *PyMOL*. The atomic coordinates of the H2A nucleosome were obtained from the human H2A nucleosome structure (PDB entry 3afa). The *B* factors of the H2A.Z.1 and H2A.Z.2 molecules in the nucleosomes were calculated using *PHENIX*. The *B* factors of the H2A nucleosome used for comparison were calculated from the canonical nucleosome structure (PDB entry 3afa).

### 2.4. Salt-resistance assay

The nucleosomes containing H2A, H2A.Z.1 or H2A.Z.2 were incubated at 328 K for 1 h in 20 m*M* Tris–HCl pH 7.5 buffer containing 1 m*M* EDTA, 1 m*M* DTT and 400, 600, 700 or 800 m*M* NaCl. After the reaction, the NaCl concentration was adjusted to 400 m*M* and 5% sucrose was added to the samples. The samples were analyzed by nondenaturing 6% PAGE with ethidium bromide staining.

### 2.5. Fluorescence recovery after photobleaching (FRAP) analysis

DNA fragments encoding histones H2A, H2A.Z.1, H2A.Z.2, H2A.Z.1 S38T and H2A.Z.2 T38S were cloned into the pEGFP-C3 vector, which encodes a GFP tag for fusion at the N-terminus. HeLa cells were transfected with the GFP-­histone vectors using Lipofectamine 2000 (Invitrogen) according to the manufacturer’s instructions. Cells stably expressing either GFP-H2A, H2A.Z.1, H2A.Z.2, H2A.Z.1 S38T or H2A.Z.2 T38S were selected in 1 mg ml^−1^ G418 (Nacalai Tesque). To measure the expression-level range of the individual GFP-histones, the fluorescent intensities of about 200 cells were obtained using a fluorescence microscope (Ti-E; Nikon) and were analyzed using the *NIS* software (Nikon). FRAP was performed on cells with similar expression levels (*i.e.* fluorescence intensities) among the different cell lines in the presence of 100 µg ml^−1^ cycloheximide using a confocal microscope (FV-1000; Olympus) with a 60× UPlanSApo numerical aperture 1.35 lens. One confocal image of a field containing 4–6 nuclei was collected (512 × 512 pixels, zoom 2, scan speed 2 µs per pixel, 800 µm pinhole, Kalman filtration for four scans, LP505 emission filter and 0.1% transmission of a 488 nm Ar laser), one half of each nucleus was bleached using 100% transmission of 488 nm (three iterations) and images were obtained using the original setting at 5 min intervals. The fluorescence intensity of the bleached area was measured using *ImageJ* 1.46r (W. Rasband; http://rsb.info.nih.gov/ij/). After background subtraction, the intensity was normalized to the intensity of the unbleached region.

## 3. Results

### 3.1. Structural differences in the L1 loop regions of H2A.Z.1 and H2A.Z.2

**Figure 1:**
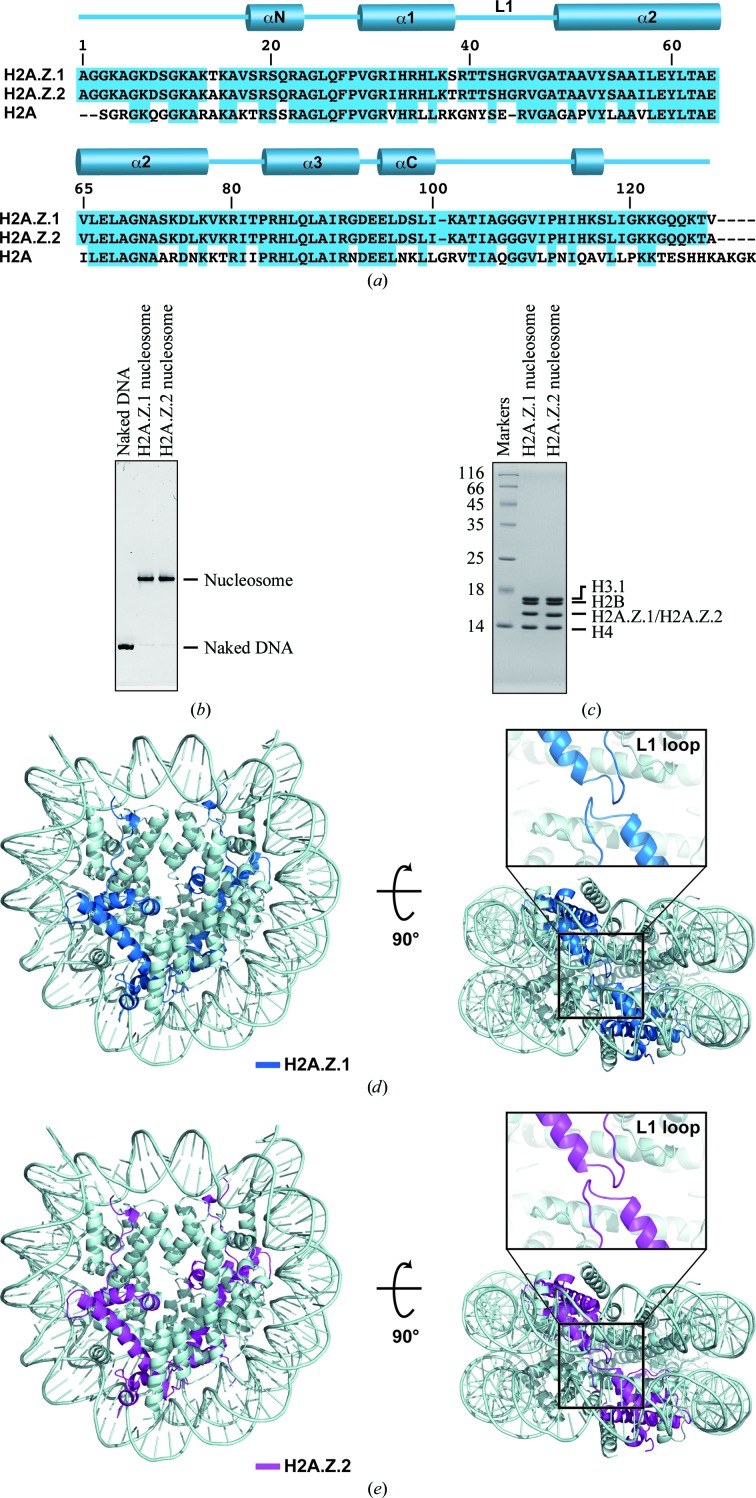
Crystal structures of human nucleosomes containing histone H2A.Z.1 or H2A.Z.2. (*a*) Alignment of the human H2A, H2A.Z.1 and H2A.Z.2 amino-acid sequences. The secondary structure of H2A.Z in the nucleosome is shown at the top of the panel. Amino-acid residues that differ among H2A.Z.1, H2A.Z.2 and H2A are represented with a white background. (*b*) Purified nucleosomes containing either H2A.Z.1 or H2A.Z.2 were analyzed by 6% nondenaturing PAGE. DNA was visualized by EtBr staining. (*c*) The histone composition of the purified nucleosomes was analyzed by 18% SDS–PAGE. Histones were visualized by Coomassie Brilliant Blue staining. (*d*) Crystal structure of the nucleosome containing human histone H2A.Z.1. Two views are presented and the H2A.Z.1 molecules are coloured blue. The L1 loop region of H2A.Z.1 is enlarged and presented at the top of the right panel. (*e*) Crystal structure of the nucleosome containing human histone H2A.Z.2. Two views are presented and the H2A.Z.2 molecules are coloured magenta. The L1 loop region of H2A.Z.2 is enlarged and presented at the top of the right panel.

Three amino-acid differences exist between human histones H2A.Z.1 and H2A.Z.2: at amino-acid residues 14 (Thr in H2A.Z.1 and Ala in H2A.Z.2), 38 (Ser in H2A.Z.1 and Thr in H2A.Z.2) and 127 (Val in H2A.Z.1 and Ala in H2A.Z.2) (Fig. 1 ▶ *a*). Amino-acid residues 14 and 127 of H2A.Z are located in the unstructured N-terminal and C-terminal tails, respectively. On the other hand, amino-acid residue 38 is located within the histone-fold domain (Luger *et al.*, 1997 ▶; Suto *et al.*, 2000 ▶).

We purified human histones H2A.Z.1 and H2A.Z.2 as bacterially expressed recombinant proteins. Purified H2A.Z.1 and H2A.Z.2 formed dimers with histone H2B as well as with the canonical histone H2A. The histone octamers containing histones H2B, H3, H4 and either H2A.Z.1 or H2A.Z.2 in a 1:1:1:1 stoichiometry were also formed without DNA in the presence of 2 *M* NaCl. The nucleosomes containing either human histone H2A.Z.1 or H2A.Z.2 were then reconstituted by the salt-dialysis method and were purified by non­denaturing polyacrylamide gel electrophoresis using a Prep Cell apparatus (Fig. 1 ▶ *b*). The nucleosomes contained human histones H2B, H3, H4 and either H2A.Z.1 or H2A.Z.2 in a 1:1:1:1 stoichiometry (Fig. 1 ▶ *c*).

**Figure 2:**
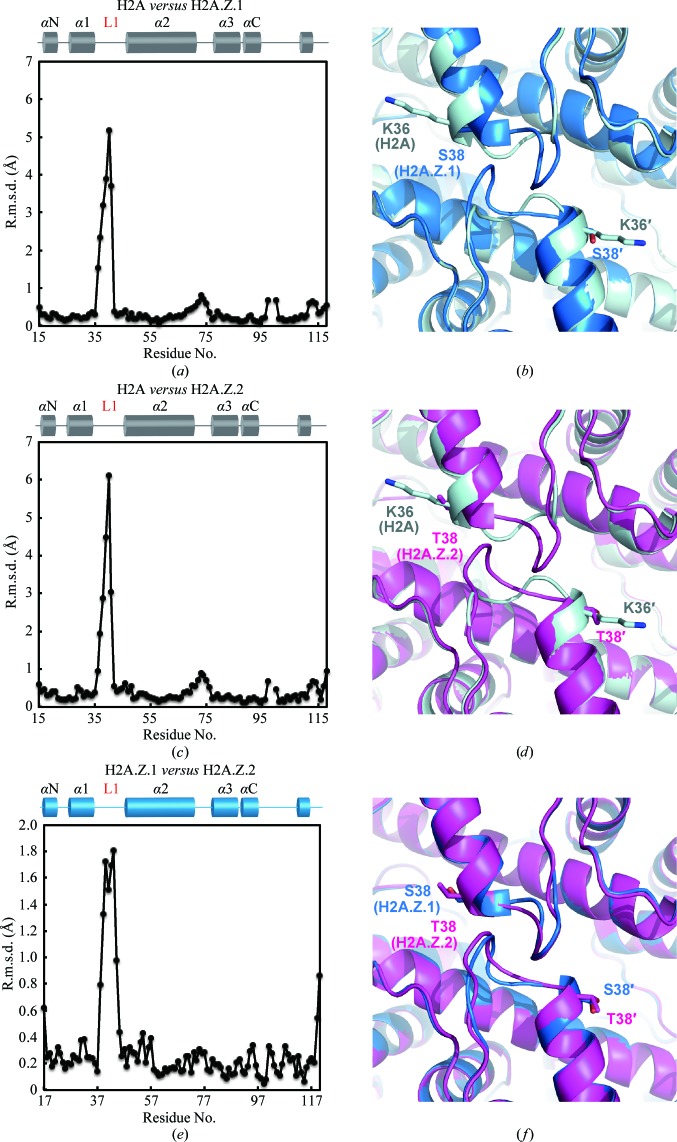
Structural comparison of nucleosomes containing H2A.Z.1, H2A.Z.2 and H2A. (*a*) Superimposition of the H2A.Z.1 nucleosome on the canonical H2A nucleosome (PDB entry 3afa; Tachiwana *et al.*, 2010 ▶). The r.m.s.d. values were calculated and plotted for each C^α^-atom pair of H2A.Z.1 and H2A. Residue numbers correspond to H2A. The r.m.s.d. value for position 98 is missing because the corresponding residue is absent in H2A.Z.1. The secondary structure of H2A in the nucleosome is shown at the top of the panel. (*b*) A close-up view around the L1 loop regions of H2A.Z.1 (blue) and H2A (grey) in the nucleosomes. The H2A.Z.1 Ser38 and H2A Lys36 residues, which are located just before the L1 loop, are represented with their side chains. (*c*) Superimposition of the H2A.Z.2 nucleosome on the canonical H2A nucleosome. The r.m.s.d. values were calculated and plotted for each C^α^-atom pair of H2A.Z.2 and H2A. Residue numbers correspond to H2A. The r.m.s.d. value for position 98 is missing because the corresponding residue is absent in H2A.Z.2. The secondary structure of H2A in the nucleosome is shown at the top of the panel. (*d*) A close-up view around the L1 loop regions of H2A.Z.2 (magenta) and H2A (grey) in the nucleosomes. The H2A.Z.2 Thr38 and H2A Lys36 residues, which are located just before the L1 loop, are represented with their side chains. (*e*) Superimposition of the H2A.Z.1 nucleosome on the H2A.Z.2 nucleosome. The r.m.s.d. values were calculated and plotted for each C^α^-atom pair of H2A.Z.1 and H2A.Z.2. Residue numbers correspond to H2A.Z.1 or H2A.Z.2. The secondary structure of H2A.Z.1 or H2A.Z.2 in the nucleosome is shown at the top of the panel. (*f*) A close-up view around the L1 loop regions of H2A.Z.1 (blue) and H2A.Z.2 (magenta) in the nucleosomes. The H2A.Z.1 Ser38 and H2A.Z.2 Thr38 residues, which are located just before the L1 loop, are represented with their side chains.

We determined the crystal structures of the human nucleosomes containing H2A.Z.1 or H2A.Z.2 (Figs. 1 ▶ *d* and 1 ▶ *e* and Table 1 ▶). To compare the H2A.Z.1 and H2A.Z.2 structures with the canonical H2A structure in nucleosomes, the H2A.Z.1 or H2A.Z.2 structures were separately superimposed on the canonical H2A structure (Figs. 2 ▶ *b*, 2 ▶ *d* and 2 ▶ *f*) and the r.m.s.d. for each residue pair was calculated and plotted (Figs. 2 ▶ *a*, 2 ▶ *c* and 2 ▶ *e*). Consistent with the previous structural analysis of mouse H2A.Z.1 in combination with *Xenopus laevis* H2B, H3 and H4 (Suto *et al.*, 2000 ▶), significant deviations were found in the H2A.Z1 L1 loop regions (amino-acid residues 39–48; Figs. 2 ▶ *a* and 2 ▶ *b*), indicating that structural differences exist between human H2A.Z.1 and canonical H2A in the L1 loop region. The L1 loop structure of H2A.Z.2 was also different from that of canonical H2A (Figs. 2 ▶ *c* and 2 ▶ *d*). Substantial structural deviations exist between H2A.Z.1 and H2A.Z.2 in the L1 loop regions, although the amino-acid sequences of both L1 loops are identical (Figs. 2 ▶ *e* and 2 ▶ *f*). This polymorphism may be caused by the amino-acid substitution at position 38 (Figs. 1 ▶ *a* and 2 ▶ *f*). The H2A.Z.1 Ser38 and H2A.Z.2 Thr38 residues sit at the C-­terminal edge of the α1 helix, which is located just before the L1 loop. Therefore, this amino-acid difference may directly affect the neighbouring L1 loop structure.

### 3.2. The H2A.Z.1 and H2A.Z.2 L1 loop regions are flexible

**Figure 3:**
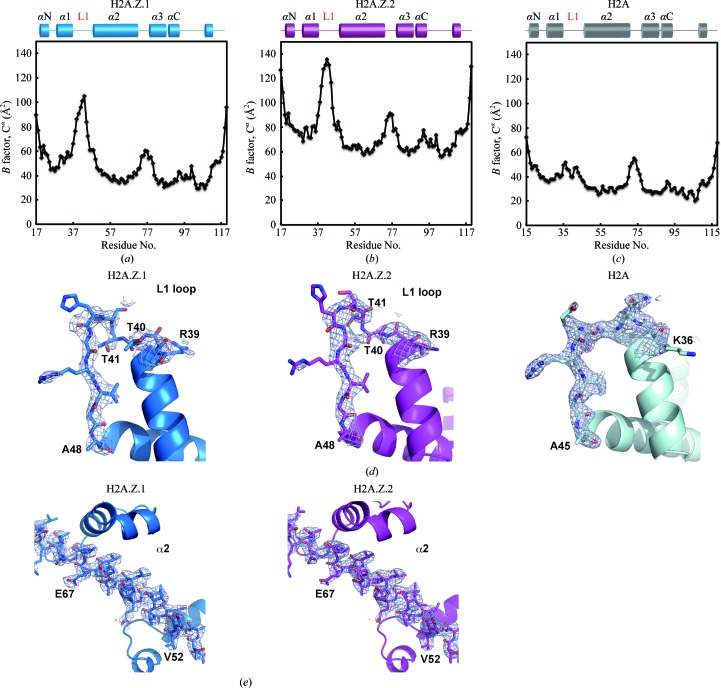
The L1 loops of H2A.Z.1 and H2A.Z.2 are flexible in nucleosomes. (*a*–*c*) The *B* factors for each C^α^ atom of H2A.Z.1 (*a*), H2A.Z.2 (*b*) and H2A (*c*) in the nucleosomes are plotted. The secondary structures of H2A.Z.1, H2A.Z.2 and H2A in the nucleosomes are shown at the top of each panel. (*d*) Close-up views of the L1 loop regions of H2A.Z.1 (left panel), H2A.Z.2 (centre panel) and H2A (right panel). The 2*mF*_o_ − *DF*_c_ maps of the L1 loop regions of H2A.Z.1, H2A.Z.2 and H2A were calculated and contoured at the 1.5σ level. (*e*) Close-up views of the α2 region of H2A.Z.1 (left panel) and H2A.Z.2 (right panel). The 2*mF*_o_ − *DF*_c_ maps of the α2 regions of H2A.Z.1 and H2A.Z.2 are presented (1.5σ level).

The structural differences between the H2A.Z.1 and H2A.Z.2 L1 loop regions may also reflect their flexible nature. Consistent with this idea, the *B* factors of the L1 loop regions of H2A.Z.1 and H2A.Z.2 are quite high compared with the surrounding region (Figs. 3 ▶ *a* and 3 ▶ *b*), unlike the L1 loop region of canonical H2A (Fig. 3 ▶ *c*). The electron densities of the side-chain moieties of the L1 loops are ambiguous in both H2A.Z.1 and H2A.Z.2 (Fig. 3 ▶ *d*, left and centre panels), although they are visible in the other H2A.Z.1 and H2A.Z.2 regions (Fig. 3 ▶ *e*) and the H2A L1 loop (Fig. 3 ▶ *d*, right panel), also suggesting high flexibility. These results indicated that the H2A.Z.1 and H2A.Z.2 L1 loop regions may be more flexible than the corresponding region of the canonical H2A.

### 3.3. Human H2A.Z.1 and H2A.Z.2 exhibit different mobilities in living cells

**Figure 4:**
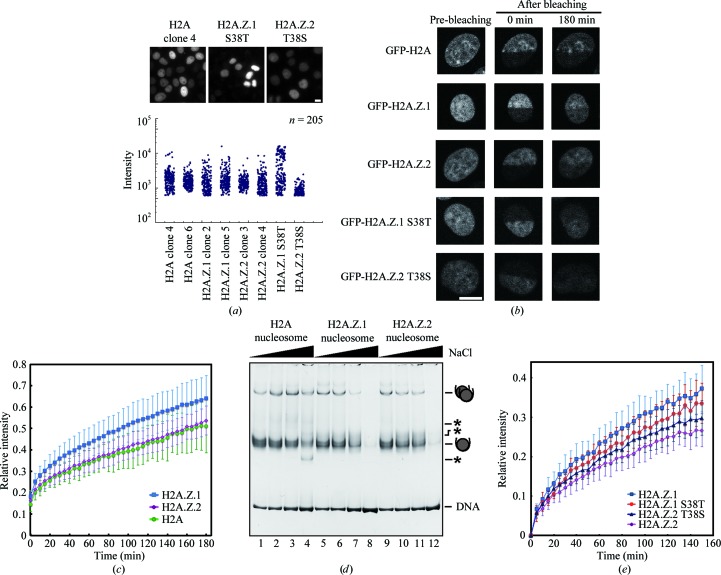
The mobilities of H2A.Z.1 and H2A.Z.2 are different in HeLa cells. (*a*) GFP-H2A.Z.1, GFP-H2A.Z.2, GFP-H2A.Z.1 S38T, GFP-H2A.Z.2 T38S and GFP-H2A were stably expressed in HeLa cells. Fluorescence images of HeLa cells stably expressing GFP-H2A (clone 4), GFP-H2A.Z.1 S38T and GFP-H2A.Z.2 T38S are presented in the upper panels. The scale bar indicates 10 µm. The lower panel shows the distribution of the fluorescence intensities of GFP-H2A (clones 4 and 6), GFP-H2A.Z.1 (clones 2 and 5), GFP-H2A.Z.2 (clones 3 and 4), GFP-H2A.Z.1 S38T and GFP-H2A.Z.2 T38S represented in arbitrary units. (*b*) HeLa cells expressing GFP-H2A.Z.1, GFP-H2A.Z.2, GFP-H2A, GFP-H2A.Z.1 S38T and GFP-H2A.Z.2 T38S were subjected to FRAP analysis. The mobility of GFP-histones in living cells was analyzed by bleaching one-half of the nucleus in the presence of 100 µg ml^−1^ cycloheximide. Representative images before bleaching (left column), upon bleaching (0 min, centre column) and 180 min after bleaching (right column) are shown. The images for GFP-H2A, GFP-H2A.Z.1 and GFP-H2A.Z.2 are presented in the top, middle and bottom rows, respectively. The scale bar indicates 10 µm. (*c*) The average relative fluorescence intensities of the bleached areas were plotted with their standard deviations (*n* = 11–36). The FRAP curves of GFP-H2A.Z.1, GFP-H2A.Z.2 and GFP-H2A are presented in blue, magenta and green, respectively. (*d*) Salt-resistance assay. The H2A nucleosomes (lanes 1–4), H2A.Z.1 nucleosomes (lanes 5–8) or H2A.Z.2 nucleosomes (lanes 9–12) were incubated in the presence of 0.4 *M* (lanes 1, 5 and 9), 0.6 *M* (lanes 2, 6 and 10), 0.7 *M* (lanes 3, 7 and 11) and 0.8 *M* NaCl (lanes 4, 8 and 12) at 328 K for 1 h. The samples were then analyzed by nondenaturing 6% PAGE with ethidium bromide staining. Bands corresponding to nucleosome monomers and nucleosome–nucleosome aggregates are indicated. Asterisks represent bands corresponding to non-nucleosomal DNA–histone complexes. (*e*) FRAP analysis of the H2A.Z.1 S38T and H2A.Z.2 T38S mutants. The average relative fluorescence intensities of the bleached areas were plotted with the standard deviations (*n* = 10–15). The FRAP curves of GFP-H2A.Z.1 S38T, GFP-H2A.Z.2 T38S, GFP-H2A.Z.1 and GFP-H2A.Z.2 are presented in dark blue, red, blue and magenta, respectively.

We next examined the mobilities of H2A.Z.1 and H2A.Z.2 as GFP-fusion proteins in living cells by fluorescence recovery after photobleaching (FRAP; Kimura, 2005 ▶). The canonical H2A tagged with GFP was used as a control. HeLa cells stably expressing GFP-fused H2A (clones 4 and 6), H2A.Z.1 (clones 2 and 5) or H2A.Z.2 (clones 3 and 4) were generated. The fluorescence intensity measurements indicated that the expression levels among the different GFP-histones were similar (Fig. 4 ▶ *a*), and cells showing similar fluorescence intensities were used for the FRAP experiments. As previously reported, the fluorescence of GFP-H2A in the bleached area recovered slowly, consistent with its incorporation into nucleosomes in living cells (Figs. 4 ▶ *b* and 4 ▶ *c*; Kimura & Cook, 2001 ▶; Gautier *et al.*, 2004 ▶; Bönisch *et al.*, 2012 ▶). Interestingly, the fluorescence recovery of GFP-H2A.Z.1 was substantially faster than that of GFP-H2A (Figs. 4 ▶ *b* and 4 ▶ *c*). This suggested that in living cells the nucleosomal H2A.Z.1 is more rapidly exchanged than the canonical H2A. Consistently, a salt-resistance assay revealed that the reconstituted H2A.Z.1 nucleosome was unstable compared with the canonical H2A nucleosome (Fig. 4 ▶ *d*). However, the fluorescence recovery of GFP-H2A.Z.2 was almost the same as that of GFP-H2A (Figs. 4 ▶ *b* and 4 ▶ *c*), although the stability of the reconstituted H2A.Z.2 nucleosome was clearly different from that of the canonical H2A nucleosome (Fig. 4 ▶ *d*). Therefore, the mobilities of H2A.Z.1 and H2A.Z.2 may be independently regulated in living cells, probably by histone chaperones and/or nucleosome re­modellers that are specific for H2A.Z.1 and H2A.Z.2.

Since amino-acid residue 38 may induce the structural polymorphism in the L1 loops, we suspected that the amino-acid difference at this position may also be responsible for the different mobilities of H2A.Z.1 and H2A.Z.2 in living cells. Therefore, we also generated cells stably expressing the GFP-H2A.Z.1 S38T and GFP-H2A.Z.2 T38S mutants, in which H2A.Z.1 Ser38 and H2A.Z.2 Thr38 were replaced by Thr and Ser, respectively (Fig. 4 ▶ *a*). Cells showing similar fluorescence intensities to the wild-type GFP-histones were used in FRAP experiments (Fig. 4 ▶ *a*). Interestingly, the fluorescence recovery of GFP-H2A.Z.1 S38T was obviously slower than that of wild-­type GFP-H2A.Z.1 (Fig. 4 ▶ *e*). In contrast, the fluorescence recovery of GFP-H2A.Z.2 T38S became faster than that of wild-type GFP-H2A.Z.2 (Fig. 4 ▶ *e*). These results suggested that the structural changes in the L1 loop induced by the different amino-acid residues at position 38 may be at least partially responsible for the different H2A.Z mobilities in cells. As the amino-acid swapping at amino-acid residue 38 did not fully convert the mobility to that of the other variant, the differences at the N-terminal and C-terminal tails (*i.e.* amino-acid residues 14 and 127) may also affect the mobility in cells, probably through distinctive post-translational modifications and/or interactions with regulatory proteins.

## 4. Discussion

Although H2A.Z is involved in many biological processes, most of the previous studies related to H2A.Z did not describe the individual functions of H2A.Z.1 and H2A.Z.2. Genetic studies of mouse H2A.Z.1 and chicken H2A.Z.1 and H2A.Z.2 suggested that they have distinct functions (Faast *et al.*, 2001 ▶; Matsuda *et al.*, 2010 ▶). Human H2A.Z.1 and H2A.Z.2 differ by only three amino acids (Eirín-López *et al.*, 2009 ▶). However, the means by which these amino-acid substitutions affect the structure and stability of nucleosomes containing H2A.Z.1 and H2A.Z.2 have not been elucidated. In this study, we present the first evidence for differences between H2A.Z.1 and H2A.Z.2 in terms of their structures and mobilities.

H2A.Z reportedly forms a heterotypic nucleosome which contains one H2A.Z and one canonical H2A within the same nucleosome (Chakravarthy *et al.*, 2004 ▶; Viens *et al.*, 2006 ▶; Nekrasov *et al.*, 2012 ▶). However, a previous structural study with mouse H2A.Z.1 suggested that the L1 loop of H2A.Z.1 may cause a steric clash if it is accommodated together with canonical H2A in the same nucleosome (Suto *et al.*, 2000 ▶). In the present study, we found that the H2A.Z.1 and H2A.Z.2 L1 loop regions are quite flexible. These findings may explain why the L1 loops of canonical H2A and H2A.Z.1 or H2A.Z.2 do not sterically clash within the heterotypic nucleosome. The structures of the H2A.Z.1 and H2A.Z.2 L1 loops may bend to avoid a steric clash with the canonical H2A L1 loop in heterotypic nucleosomes.

In cells, histone exchange is mediated by the cooperative actions of histone chaperones (Park & Luger, 2008 ▶; Das *et al.*, 2010 ▶; Avvakumov *et al.*, 2011 ▶) and nucleosome remodellers (Alkhatib & Landry, 2011 ▶; Hargreaves & Crabtree, 2011 ▶). In yeast, the histone chaperone Chz1 and two nucleosome-remodelling complexes, INO80 and SWR1, have been identified as major factors involved in H2A.Z exchange (Shen *et al.*, 2000 ▶; Mizuguchi *et al.*, 2004 ▶; Luk *et al.*, 2007 ▶; Papamichos-Chronakis *et al.*, 2011 ▶). Since the presence of an H2A.Z.2 orthologue has not been reported in yeast, it is not known whether the vertebrate counterparts of these yeast nucleosome regulatory factors can discriminate between H2A.Z.1 and H2A.Z.2. The present study revealed that in nucleosomes within living cells H2A.Z.1 is more rapidly exchanged than H2A.Z.2. However, *in vitro* there is no obvious difference in the stabilities of the reconstituted H2A.Z.1 and H2A.Z.2 nucleosomes, as revealed by a salt-resistance assay. These results suggested that the mobilities of H2A.Z.1 and H2A.Z.2 *in vivo* may be regulated by their specific histone chaperones and/or nucleosome remodellers. In the future, it will be intriguing to search for the specific factors regulating H2A.Z.1 and H2A.Z.2.

H2A.Z is predominantly located around the promoters of active genes and is suggested to regulate transcription initiation (Guillemette *et al.*, 2005 ▶; Albert *et al.*, 2007 ▶; Barski *et al.*, 2007 ▶; Creyghton *et al.*, 2008 ▶; Zilberman *et al.*, 2008 ▶; Cui *et al.*, 2009 ▶; Hardy *et al.*, 2009 ▶; Conerly *et al.*, 2010 ▶; Kelly *et al.*, 2010 ▶; Weber *et al.*, 2010 ▶; Soboleva *et al.*, 2011 ▶; Valdés-Mora *et al.*, 2012 ▶; Nekrasov *et al.*, 2012 ▶; Li *et al.*, 2012 ▶; Hu *et al.*, 2013 ▶). In human and chicken cells, comparable amounts of H2A.Z.1 and H2A.Z.2 are expressed (Dryhurst *et al.*, 2009 ▶; Matsuda *et al.*, 2010 ▶). Our FRAP analysis revealed that H2A.Z.1 exchanged more rapidly than H2A.Z.2 in living cells. Therefore, incorporation of H2A.Z.1 and H2A.Z.2 at the promoters may induce different outcomes in transcriptional regulation.

In addition, H2A.Z functions with HP1 to form heterochromatin, in which transcription is generally repressed (Rangasamy *et al.*, 2004 ▶; Fan *et al.*, 2004 ▶). H2A.Z.1 and H2A.Z.2, which have different mobilities in cells, may be important in the selective formation of active and inactive chromatin architectures. Further studies are required to clarify this issue.

## Supplementary Material

PDB reference: H2A.Z.1 nucleosome, 3wa9

PDB reference: H2A.Z.2 nucleosome, 3waa

## Figures and Tables

**Table 1:** Data-collection and refinement statistics Values in parentheses are for the highest resolution shell.

	H2A.Z.1 nucleosome	H2A.Z.2 nucleosome
Data collection		
Space group	*P*2_1_2_1_2_1_	*P*2_1_2_1_2_1_
Unit-cell parameters (Å)	*a* = 104.90, *b* = 109.39, *c* = 181.76	*a* = 105.36, *b* = 109.84, *c* = 182.99
Resolution (Å)	50.0–3.07 (3.18–3.07)	50.0–3.20 (3.31–3.20)
No. of reflections	2790970	1511021
No. of unique reflections	40613	35825
Completeness (%)	99.8 (100)	98.3 (99.4)
*R*_merge_ † (%)	7.9 (48.9)	9.9 (49.4)
〈*I*/σ(*I*)〉	12.3 (4.5)	9.3 (3.3)
Multiplicity	6.3 (5.9)	4.5 (4.4)
Refinement		
Resolution (Å)	39.0–3.07	39.2–3.20
*R*_work_/*R*_free_ ‡ (%)	22.2/27.1	21.7/27.1
*B* factors (Å^2^)		
Protein	45.5	72.3
DNA	101.5	124.8
R.m.s. deviations		
Bond lengths (Å)	0.008	0.009
Bond angles (°)	1.32	1.36
Ramachandran favoured (%)	97.0	96.1
Ramachandran outliers (%)	0.0	0.0
PDB code	3wa9	3waa

† *R*_merge_ = 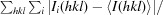 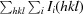.

‡ *R*_work_ = 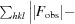 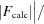 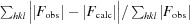. *R*_free_ was calculated with 5% of the data excluded from the refinement.
